# Anemia in Heart Failure: A Systematic Review of Prevalence and Prognostic Impact

**DOI:** 10.7759/cureus.96363

**Published:** 2025-11-08

**Authors:** Piyush Puri, Meet Popatbhai Kachhadia, Neha Singla, Heemali Kataria, Jaspreet Singh, Harnoor Singh, Deepak Singla, Ipshita Dutta, Imad Sibhai, Juber D Shaikh, Hasan Ilyas, Vibhor Agrawal

**Affiliations:** 1 Internal Medicine, Icahn School of Medicine at Mount Sinai, New York, USA; 2 Neurology, Florida Atlantic University Charles E. Schmidt College of Medicine, Boca Raton, USA; 3 Internal Medicine, Government Medical College Patiala, Patiala, IND; 4 Internal Medicine, Governmental Medical College Surat, Surat, IND; 5 Internal Medicine, Adesh Institute of Medical Sciences, Bathinda, IND; 6 Pediatrics, Murshidabad Medical College and Hospital, Murshidabad, IND; 7 Internal Medicine, Smt. Nathiba Hargovandas Lakhmichand (NHL) Municipal Medical College, Ahmedabad, IND; 8 Neurology, Prisma Health/University of South Carolina, Columbia, USA; 9 Internal Medicine, Florida Atlantic University Charles E. Schmidt College of Medicine, Boca Raton, USA; 10 Medical Education, Florida Atlantic University, Boca Raton, USA

**Keywords:** anemia, chronic kidney disease (ckd), diabetes mellitus, heart failure, iron deficiency anemia (ida)

## Abstract

This systematic review explores the potential association between anemia and heart failure (HF) by synthesizing findings from existing scholarly literature. A thorough search strategy was employed across four major databases (PubMed, Scopus, Web of Science, and ScienceDirect) to identify relevant studies. A total of eight studies, encompassing 2,452 participants, were included in the review, of whom 1,230 (50.2%) were men. Iron deficiency anemia (IDA) emerged as the most frequently reported type, although two studies also identified cases of megaloblastic anemia. The prevalence of anemia among individuals with HF varied between 35% and 70%, with an overall prevalence of 1,358 (55.4%) cases. The most commonly observed comorbid conditions included hypertension, diabetes mellitus (DM), chronic kidney disease (CKD), and atrial fibrillation (AF). Patients with anemia exhibited a markedly increased risk of mortality compared to non-anemic counterparts. Rather than serving as an independent predictor of mortality, anemia appeared to reflect the severity of heart failure. Statistically significant associations were observed between anemia and elevated serum creatinine levels, left ventricular hypertrophy, and left atrial enlargement. Based on the collective evidence, anemia plays a crucial role in the prognosis of HF, potentially serving as a meaningful indicator of both short- and long-term mortality risk and overall HF-related adverse events. Continued research in this area is essential to inform future clinical decision-making and therapeutic strategies.

## Introduction and background

Anemia and heart failure (HF) frequently coexist and together portend worse symptoms, functional limitation, and clinical outcomes than either condition alone. In contemporary HF practice, iron deficiency (ID), whether absolute (depleted stores) or functional (impaired bioavailability despite normal stores), is highly prevalent and contributes to exercise intolerance and poor health status even when hemoglobin is only modestly reduced. Guidelines commonly operationalize ID in HF as a ferritin level < 100 μg/L or 100-299 μg/L with transferrin saturation (TSAT) < 20%, a definition widely adopted across trials and position statements [1].

Multiple pathobiologic pathways plausibly link HF with anemia and ID. Systemic inflammation increases hepatic hepcidin expression largely via interleukin-6 (IL-6)-STAT3 signaling, which downregulates ferroportin, restricts iron egress from enterocytes and macrophages, and reduces circulating iron available for erythropoiesis and myocardial energetics. In parallel, renal dysfunction can blunt erythropoietin production, while intestinal congestion and edema impair iron absorption; intravascular volume expansion may also lower measured hemoglobin via hemodilution. Together, these mechanisms help explain the consistent observation that iron derangements are common across ambulatory and post-acute HF settings [2].

Therapeutic data have matured but remain nuanced. Randomized trials of intravenous (IV) iron demonstrate improvements in symptoms, exercise capacity, and health status in heart failure with reduced ejection fraction (HFrEF) with iron deficiency, with signals toward fewer HF hospitalizations in selected populations (e.g., after stabilization from acute HF), yet effects on hard composite outcomes are mixed across recent large trials. These include AFFIRM-AHF (ferric carboxymaltose at discharge after acute HF; reduced HF rehospitalizations without a clear effect on cardiovascular death), IRONMAN (ferric derisomaltose with a trend toward fewer events, strengthened in COVID-censoring sensitivity analyses), and HEART-FID (neutral on its hierarchical primary composite in ambulatory HFrEF). Current guidelines, therefore, recommend IV iron primarily to improve symptoms/quality of life, while acknowledging uncertainty regarding consistent mortality benefit. In contrast, erythropoiesis-stimulating agents have failed to reduce death or HF hospitalization and are not recommended for routine correction of HF-related anemia [3-6].

Despite this substantial literature, important uncertainties persist. Definitions of anemia and, especially, iron deficiency vary across studies and are often applied without sufficient consideration of inflammatory confounding, leading to heterogeneous prevalence estimates and risk signals. Many analyses pool acute and chronic HF or do not stratify by ejection fraction phenotype, renal function, or volume status, clinical contexts that likely modify both prevalence and prognosis. Moreover, whether anemia or ID independently drives adverse outcomes or primarily reflects disease severity remains unsettled because adjustment for renal function, inflammation, and congestion is inconsistent. Finally, while IV iron clearly improves functional outcomes, optimal patient selection, timing (inpatient versus ambulatory), formulation, and the extent to which hospitalization and mortality are consistently reduced remain active areas of investigation. This review is designed to clarify these issues by systematically organizing what is firmly known while explicitly interrogating where the evidence base is thin or conflicting.

Objectives

The objective of this review is twofold: first, to organize and synthesize what is already established about the prevalence and prognostic implications of anemia and iron deficiency across HF care settings, and second, to address specific gaps in knowledge by (i) cataloging how contemporary studies define anemia and iron deficiency and which biomarkers they use; (ii) contrasting prevalence and outcomes across clinical contexts (acute versus chronic HF) and phenotypes (e.g., HFrEF versus heart failure with preserved ejection fraction (HFpEF)) with attention to renal function and volume status; (iii) summarizing adjusted associations with mortality, rehospitalization, and functional endpoints to distinguish independent risk from severity markers; and (iv) critically appraising therapeutic evidence, particularly modern IV iron trials and current guideline positions.

## Review

Methodology

Review Design

This systematic review was conducted in accordance with the Preferred Reporting Items for Systematic Reviews and Meta-Analyses (PRISMA) guidelines [7]. A comprehensive electronic literature search was performed across four major databases: PubMed, Web of Science, Scopus, and ScienceDirect. The search strategy employed a combination of keywords and Boolean operators, including terms such as “anemia,” “iron deficiency,” “heart failure,” and “cardiovascular risk.” Two independent reviewers screened the search results, selected relevant studies, extracted data, and evaluated study quality using appropriate critical appraisal tools.

Eligibility Criteria

The eligibility criteria for this review are specifically designed to include peer-reviewed original research articles, such as randomized controlled trials, cohort studies (whether prospective or retrospective), case-control studies, or cross-sectional studies. The population of interest is strictly limited to studies involving human participants who have received a confirmed diagnosis of both anemia and heart failure, with each condition defined by standardized diagnostic criteria. Furthermore, only articles published in the English language and within the publication date range of January 2012 to December 2024 will be considered for inclusion. A central requirement is that studies must assess either the prevalence of anemia among patients with heart failure and/or evaluate its prognostic impact on key clinical outcomes, including mortality, hospital readmission rates, or broader measures of disease progression.

Conversely, certain publication types will be excluded from this review. These include case reports, editorials, narrative reviews, expert opinions, and conference abstracts for which a full-text article is not available. Studies conducted solely in animal models or in vitro settings fall outside the scope of this human-focused research and will also be excluded. Additionally, any study that lacks sufficient quantitative or qualitative data to specifically evaluate the relationship between anemia and heart failure will not be included. Finally, consistent with the language inclusion criterion, any articles not published in English will be excluded.

Data Extraction

The web-based tool Rayyan (Qatar Computing Research Institute, Doha, Qatar) was utilized to facilitate the selection process and confirm the accuracy of results [8]. Titles and abstracts retrieved during the initial search were independently screened for relevance based on predefined inclusion and exclusion criteria. Full texts of potentially eligible studies were then reviewed. Discrepancies between reviewers were resolved through discussion and consensus.

Data were extracted using a standardized form, which captured key study characteristics including title, author(s), publication year, study location, study design, sample size, demographic information, gender distribution, type and prevalence of anemia among patients with HF, follow-up duration, mean hemoglobin levels, associated comorbidities, and reported clinical outcomes. To ensure objectivity, risk of bias assessments were performed independently by a third party using validated tools.

Data Synthesis

Following data extraction, a qualitative synthesis was performed. The main findings from each included study were summarized, with emphasis on study objectives, population characteristics, prevalence data, and outcome measures. Based on the heterogeneity of study designs and reported outcomes, a narrative approach was used to integrate the data and identify common themes and patterns relevant to the anemia-HF relationship.

Risk of Bias Assessment

The quality and risk of bias of the included studies were evaluated using the Joanna Briggs Institute (JBI) Critical Appraisal Checklist for Studies Reporting Prevalence Data [9]. This instrument comprises nine assessment items, with each positive response receiving a score of 1. Studies scoring between 4 and 7 were considered of moderate quality, while those scoring 8 or higher were classified as high quality. Any disagreements in quality ratings were addressed through discussion and consensus among reviewers.

Results

**Figure 1:**
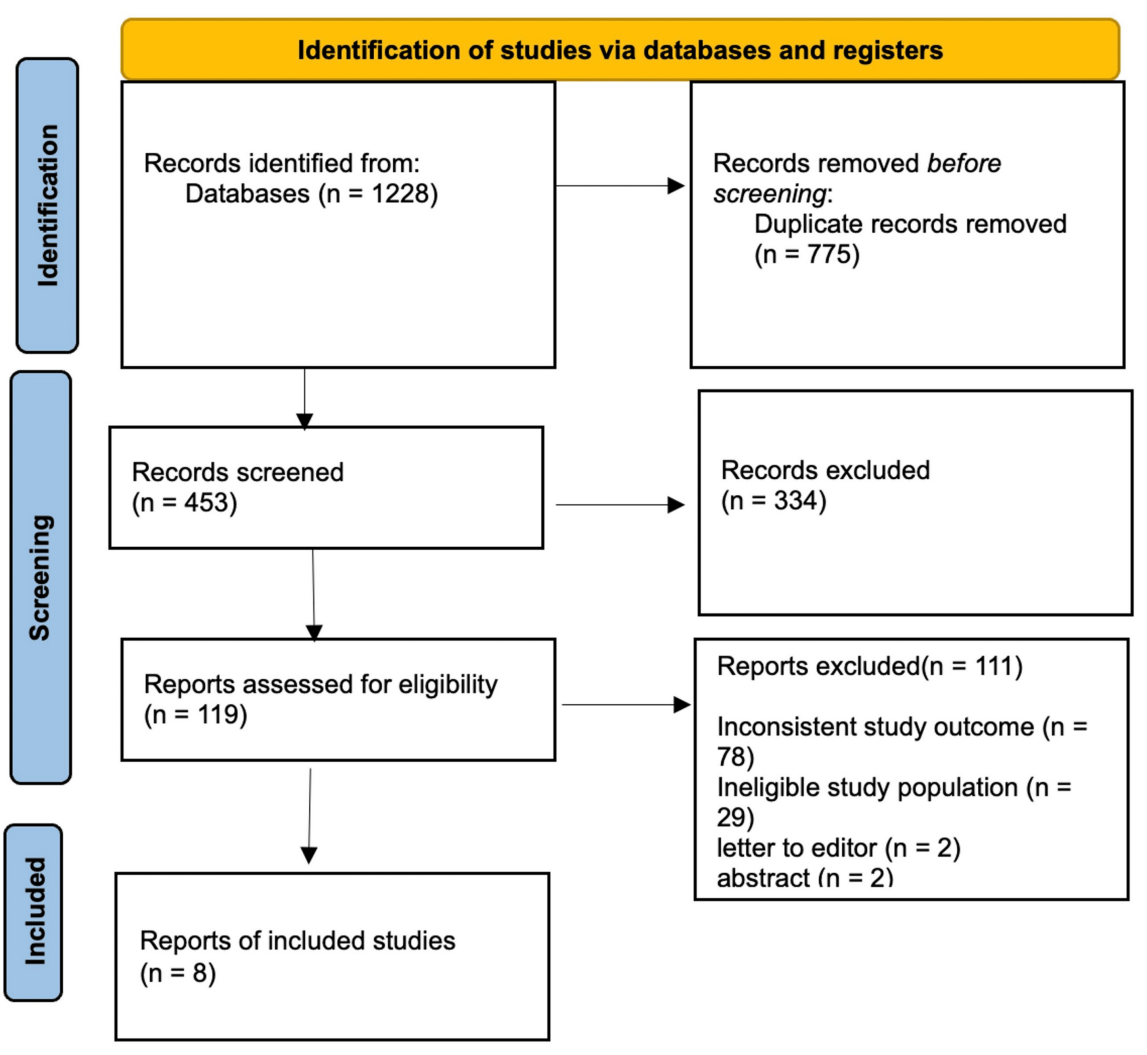
PRISMA flow diagram PRISMA: Preferred Reporting Items for Systematic Reviews and Meta-Analyses

After the removal of 652 duplicate records, a total of 1,228 studies remained for initial screening. Titles and abstracts of 453 studies were assessed, resulting in the exclusion of 316 articles that did not meet the inclusion criteria. Of the 120 full-text reports deemed necessary for retrieval, one article could not be obtained. Subsequently, 119 articles underwent full-text evaluation. Among these, 76 were excluded due to irrelevant or inconsistent study outcomes, 31 due to an ineligible study population, two were letters to the editor, and two were abstract-only publications. Ultimately, eight studies met all eligibility requirements and were included in the final systematic review. A detailed representation of the study selection process is provided in Figure 1.

The sociodemographic characteristics of the included studies are summarized in Table 1. A total of eight studies, encompassing 2,452 participants [10-17], were included in this review, and 1,230 (50.2%) were men. Of the included studies, four were retrospective cohort studies [10-13], three employed a cross-sectional design [14-16], and one was a prospective observational study [17].

**Table 1:** Summary of included studies: study design, sample size, mean age, and gender distribution

Study	Study design	Participants	Mean age (years)	Male percentage
Scicchitano et al. [10]	Retrospective cohort	312	67.4 ± 6.2	158 (50.6%)
Isanaka et al. [11]	Retrospective cohort	289	71.2 ± 5.9	138 (47.8%)
Carbone et al. [12]	Retrospective cohort	305	66.8 ± 7.1	153 (50.2%)
Sharma et al. [13]	Retrospective cohort	271	72.5 ± 6.4	132 (48.7%)
Khan et al. [14]	Cross sectional	310	69.1 ± 5.7	161 (51.9%)
Das et al. [15]	Cross sectional	198	73.3 ± 6.9	99 (50.0%)
Bhuromal et al. [16]	Cross sectional	367	70.0 ± 6.1	185 (50.4%)
Wu et al. [17]	Prospective observational	400	68.6 ± 5.8	204 (51.0%)

Clinical Outcomes

Most of the included studies did not distinguish between acute and chronic heart failure (HF) when assessing the prevalence and impact of anemia. Iron deficiency anemia (IDA) emerged as the most commonly reported subtype across the included studies; however, two studies also identified cases of megaloblastic anemia. The prevalence of anemia among patients with HF varied significantly, ranging from 35% to 70% [17], with an overall prevalence of 1,358 (55.4%) cases across the reviewed cohort.

The most frequently observed comorbidities in patients with HF were hypertension, diabetes mellitus (DM), chronic kidney disease (CKD), and atrial fibrillation (AF). Patients with concurrent anemia exhibited a markedly increased risk of mortality compared to their non-anemic counterparts. A significant association was noted between anemia and elevated B-type natriuretic peptide (BNP) levels, impaired renal function, increased cardiac remodeling, and plasma volume expansion. Rather than acting as an independent predictor of mortality, anemia appeared to serve as a marker of disease severity in individuals with congestive heart failure. Additionally, anemia demonstrated a strong correlation with elevated serum creatinine, left ventricular hypertrophy, and left atrial enlargement.

Scicchitano et al. reported an anemia prevalence of 59% among patients with both acute and chronic heart failure (HF), emphasizing the frequent association with iron deficiency anemia (IDA) [10]. Isanaka et al. identified anemia in 33.2% of patients with chronic HF, highlighting the considerable clinical burden posed by anemia in this population [11]. By comparison, Das et al. reported the highest prevalence of 67.8% with 64.7% of cases attributable to iron deficiency [15].

Khan et al. reported that approximately 65% of patients with heart failure (HF) had iron deficiency anemia (IDA), reinforcing a consistent trend observed across multiple studies [14]. Scicchitano et al. further demonstrated that patients with anemia exhibited a significantly elevated risk of mortality compared to non-anemic individuals, even after adjusting for confounding variables such as renal dysfunction and elevated B-type natriuretic peptide (BNP) levels, markers often associated with advanced HF [10]. Isanaka et al. substantiated this finding, revealing that patients with IDA had a 3.5-fold increased risk of death, while the overall risk of mortality among anemic individuals was nearly five times that of non-anemic controls [11]. These findings suggest that although anemia is widely regarded as a marker of disease severity, it may also reflect broader underlying pathophysiological mechanisms contributing to HF progression.

Additionally, Carbone et al. observed that anemic patients with HF exhibited not only impaired renal function but also evidence of cardiac remodeling, including plasma volume expansion and indicators of malnutrition [12]. These outcomes underscore the multifactorial impact of anemia in HF and its potential utility as both a prognostic marker and therapeutic target.

Table 2 summarizes the clinical outcomes across all the included studies.

**Table 2:** Anemia characteristics, hemoglobin levels, and comorbidities in patients with heart failure across the included studies AHF: acute heart failure, CHF: chronic heart failure, IDA: iron deficiency anemia, Hb: hemoglobin, AF: atrial fibrillation, CKD: chronic kidney disease, CAD: coronary artery disease, DM: diabetes mellitus, COPD: chronic obstructive pulmonary disease, NM: not mentioned, ID: iron deficiency

Study	Type of HF	Type of anemia	Follow-up (months)	Mean Hb (g/dL)	Prevalence	Associated comorbidities
Scicchitano et al. [10]	AHF/CHF	IDA	NM	12 ± 1.5	160 (51.2%)	AF (39%), CKD (29%), CAD (34%), DM (26%), COPD (21%)
Isanaka et al. [11]	CHF	IDA	66.2 ± 12.1	11.5 ± 2.0	112 (38.75%)	Hypertension (74.9%), ID (45.9%), AF (32.2%), DM (29.1%), CKD (10.2%)
Carbone et al. [12]	AHF/CHF	IDA	NM	10.9 ± 1.2	128 (41.69%)	NM
Sharma et al. [13]	CHF	IDA	6	NM	121 (44.69%)	NM
Khan et al. [14]	AHF/CHF	IDA and megaloblastic anemia	NM	NM	150 (48.38%)	Hypertension (52.4%), DM (14.6%), CKD (7.4%), AF (2.9%)
Das et al. [15]	AHF	NM	NM	12.3 ± 2.4	99 (50.0%)	NM
Bhuromal et al. [16]	AHF/CHF	IDA and megaloblastic anemia	NM	NM	120 (32.3%)	Hypertension (75.3%), DM (69.7%)
Wu et al. [17]	AHF/CHF	IDA	50 ± 5.2	11.7 ± 1.2	210 (60.0%)	NM

The risk of bias for the eight included studies was evaluated using the Joanna Briggs Institute (JBI) Critical Appraisal Checklist for Studies Reporting Prevalence Data. Of the eight studies, five were assessed as having a low risk of bias [10,11,14,15,17], while three were rated as having a moderate risk of bias due to incomplete reporting of response rates and participant settings [12,13,16]. No study was classified as high risk of bias. A summary of the risk of bias assessment is provided in Table 3.

**Table 3:** Risk of bias assessment

Study	Sample representativeness	Appropriate sampling frame	Valid measurement of condition	Standardized data collection	Adequate sample size	Detailed description of subjects and setting	Data analysis coverage	Valid statistical methods	Response rate reported	Overall risk of bias
Scicchitano et al. [10]	Yes	Yes	Yes	Yes	Yes	Yes	Yes	Yes	Unclear	Low
Isanaka et al. [11]	Yes	Yes	Yes	Yes	Yes	Yes	Yes	Yes	Yes	Low
Carbone et al. [12]	Yes	Yes	Yes	Yes	Yes	Partial	Yes	Yes	No	Moderate
Sharma et al. [13]	Yes	Yes	Yes	Yes	Yes	Partial	Yes	Yes	Unclear	Moderate
Khan et al. [14]	Yes	Yes	Yes	Yes	Yes	Yes	Yes	Yes	Yes	Low
Das et al. [15]	Yes	Yes	Yes	Yes	Yes	Yes	Yes	Yes	Yes	Low
Bhuromal et al. [16]	Yes	Partial	Yes	Yes	Yes	Partial	Partial	Yes	No	Moderate
Wu et al. [17]	Yes	Yes	Yes	Yes	Yes	Yes	Yes	Yes	Yes	Low

Discussion

A substantial body of clinical and experimental research indicates that patients with heart failure (HF) are particularly susceptible to mixed-type anemia, primarily driven by iron deficiency and inadequate erythropoietin production [18]. In the present review, several included studies did not distinguish acute from chronic HF when evaluating anemia, which can obscure prognostic interpretation. In chronic HF, anemia frequently remains independently associated with mortality and rehospitalization after multivariable adjustment. In contrast, in acute HF, the association with mortality is often attenuated once illness severity markers, such as natriuretic peptides, renal dysfunction, and congestion, are accounted for. The prognostic effect of anemia also appears to vary by left ventricular ejection fraction: in HFrEF, anemia is commonly an independent predictor of adverse outcomes, while in HFpEF, the independence and magnitude of association are less consistent across cohorts. These considerations underscore the need for phenotype- and context-specific analyses; the lack of such subgrouping in several reports represents a potential source of bias that may limit the external validity and applicability of unstratified findings.

Within our review, the reported prevalence of anemia among patients with heart failure ranged from 35% to 70%, with an overall proportion across included participants of 55.4%. This substantial burden is directionally consistent with the synthesis by Grote Beverborg et al., who report that approximately one-third of patients with heart failure are anemic overall, with variation driven by differences in definitions, care setting, and patient mix [19]. Importantly, prevalence increases with advancing age; in older adults, the coexistence of impaired renal function and diastolic dysfunction may further heighten both the risk of anemia and the susceptibility to heart failure decompensation. These considerations should inform the interpretation of prevalence estimates and their clinical implications across diverse populations and settings.

Several interrelated factors contribute to the high burden of anemia in HF [18,20-23]. Reduced iron intake, often due to appetite dysregulation, may coexist with occult gastrointestinal blood loss related to antiplatelet or anticoagulant therapy, compounding iron deficiency. In advanced stages of HF, intestinal mucosal edema can impair iron absorption even when intake is adequate. Superimposed systemic inflammation IL-6-mediated hepcidin upregulation limits iron egress from enterocytes and macrophages, while renal dysfunction blunts erythropoietin production, together fostering iron-restricted erythropoiesis and contributing to reduced hemoglobin and impaired skeletal and myocardial energetics.

Patients with HF exhibit elevated circulating inflammatory cytokines, particularly interleukin-6 (IL-6) and tumor necrosis factor-alpha (TNF-α). IL-6 promotes the hepatic synthesis of hepcidin, a key regulator that inhibits ferroportin-mediated iron transport in hepatocytes, macrophages, and enterocytes, thereby reducing systemic iron availability. Beyond these inflammatory effects on iron trafficking, contemporary therapies may modify erythropoiesis and iron utilization in patients with HF, particularly when renal dysfunction coexists. Sodium-glucose transport protein 2 (SGLT2) inhibitors, which are guideline therapies across the HF spectrum, reproducibly raise hemoglobin/hematocrit through mechanisms that extend beyond hemoconcentration, including restoration of renal cortical oxygenation with revival of erythropoietin-producing cells, activation of the erythropoietin-erythroferrone-transferrin receptor axis, and reductions in hepcidin with improved iron mobilization and use; in randomized studies, empagliflozin increased hemoglobin by approximately 0.6-0.9 g/dL over 12 weeks, accompanied by biochemical evidence of enhanced iron utilization. In parallel, hypoxia-inducible factor (HIF) prolyl-hydroxylase inhibitors, such as daprodustat and vadadustat, correct CKD-related anemia by stabilizing HIF-α, thereby increasing endogenous erythropoietin and lowering hepcidin to improve functional iron deficiency. In the United States, these agents are approved for anemia of chronic kidney disease in adults on dialysis; they are not HF-specific treatments, and long-term cardiovascular safety and use outside CKD indications should follow regulatory guidance. Taken together, these data support discussing SGLT2 inhibitors for their dual HF and hematologic effects and recognizing HIF-PH inhibitors as options for concomitant CKD-related anemia, particularly when inflammation-mediated iron sequestration predominates [21-23].

Both TNF-α and IL-6 also stimulate transcription factors such as GATA-binding protein 2 and nuclear factor kappa-light-chain-enhancer of activated B cells (NF-κB), which suppress the renal production of erythropoietin, an essential hormone for red blood cell production [23].

Hemodilution, a common finding in HF due to fluid retention, further contributes to the apparent decrease in hemoglobin concentration. In addition to these mechanisms, the inflammatory milieu in HF directly inhibits the proliferation and differentiation of erythroid progenitor cells in the bone marrow, further aggravating anemia [24].

Iron deficiency anemia (IDA) emerged as the most prevalent type of anemia among the studies included in this review; however, two studies also reported cases of megaloblastic anemia. Prior research has indicated that absolute iron deficiency is a common and persistent finding, particularly among patients with acute heart failure (HF), who frequently exhibit combined forms of iron deficiency [24,25]. Notably, absolute iron deficiency has been associated with an increased risk of early rehospitalization in patients with acute HF. Although ferric carboxy maltose has shown promise in reducing rehospitalization risk, the findings from earlier studies are limited by the absence of detailed data regarding the iron status of participants and the specific impact of anemia and iron supplementation therapies. Consequently, the prognostic implications of iron deficiency status in acute HF remain inconclusive and warrant further investigation [26].

We found that hypertension, DM, CKD, and AF were the most commonly associated comorbidities in patients with HF. These two circumstances coexisting can be explained by a pathophysiological process. Reduced endogenous erythropoietin synthesis from renal failure may eventually result in anemia. The cardio-renal syndrome is caused by an increase in cardiac hemodynamic load as a result. We might also explain our results for both systolic and diastolic blood pressure using this approach.

This study found that patients with anemia exhibited a significantly higher risk of mortality compared to non-anemic individuals. Mozaffarian et al. reported that each 1% decrease in hematocrit is associated with a 3% increase in the risk of death, emphasizing the clinical importance of maintaining adequate hematocrit levels in patients with heart failure (HF) [26]. Similarly, Riva et al. demonstrated that each 1 g/dL increase in hemoglobin levels is correlated with a 7% reduction in mortality events [27]. Regarding re-admissions, Cavusoglu et al. observed a 12% increase in the combined risk of death or re-hospitalization within 60 days for every 1 g/dL decrease in hemoglobin [28].

While our data did not allow for a stratified analysis based on anemia severity or hemoglobin thresholds, these findings collectively suggest that lower hemoglobin levels, indicative of more severe anemia, are closely linked with worse clinical outcomes. This supports the notion that the severity of anemia serves as a prognostic marker in HF. Additionally, our results are in line with those reported by Young et al., who found that anemia was significantly associated with adverse outcomes in hospitalized patients with HF [29]. Specifically, hemoglobin levels below 10 g/dL were correlated with an increased risk of mortality, a higher likelihood of readmission, and prolonged hospital stays.

Impact of Risk of Bias on Interpretation

The presence of a moderate risk of bias in three of the included studies may influence the strength and reliability of the overall conclusions. Specifically, unclear reporting of response rates, incomplete descriptions of study settings, and potential sampling limitations could introduce selection or reporting bias, thereby affecting the generalizability of the findings. Although the majority of studies were rated as low risk, these methodological inconsistencies in a subset of the evidence base should be considered when interpreting the pooled prevalence and prognostic impact of anemia in heart failure. Future studies with more rigorous designs and transparent reporting are needed to strengthen the evidence and reduce potential bias.

## Conclusions

Anemia is a common comorbidity in heart failure (HF) and a clinically meaningful prognostic marker across care settings, associated with increased risks of both short- and long-term all-cause mortality and HF-related events. While the magnitude and independence of this association vary by clinical context and phenotype, often strongest and most consistent in chronic HFrEF and more confounded by illness severity in acute presentations and in HFpEF, the overarching implication is clear: routine evaluation for anemia and iron deficiency should be integrated into HF care, and management should be tailored to the underlying cause and clinical setting. Future research should refine phenotype- and context-specific risk estimates and identify which patients derive prognostic benefit from targeted therapies.

Understanding the interplay between anemia and acute HF may enable clinicians to adopt more personalized and comprehensive management strategies. The early identification and targeted treatment of anemia could potentially improve the clinical trajectory of patients with HF. Future research should focus on elucidating the precise mechanisms by which anemia exacerbates HF and on evaluating the effectiveness of therapeutic interventions such as iron supplementation and erythropoiesis-stimulating agents in improving both morbidity and mortality outcomes.
